# Supplementary material for: Mapping Community Priorities for Local Medical Centers: An Importance-Performance Analysis Study of Residents’ Perceptions in Large Cities, Non-Large Cities, and Rural Areas in South Korea
Source: Healthcare (Basel). 2025 Oct 3;13(19):2513. doi: 10.3390/healthcare13192513 (PMC12525427; doi:10.3390/healthcare13192513)
Supplement: Supplementary file 1 [file healthcare-13-02513-s001.zip › healthcare-3832789-supplementary.pdf]

Supplementary Table S1. One-way ANOVA Results for Regional Importance-Performance Gaps.

Residential Area	N	Mean (CI)	SD	F(p)
Large city	624	1.55(1.45,1.66)	1.3	
Non-large city	990	0.90(0.82,0.98)	1.3	44.510***
Rural area	443	1.30(1.15,1.45)	1.6	

SD=Standard Deviation; F=F-ratio; p=p-value.

CI = 95% Confidence Interval (Lower, Upper)

***p < .001.

Supplementary Table S2-1. Bootstrapped mean importance and performance scores with 95% bias-corrected and accelerated (BCa) confidence intervals for nine core functions of Local Medical Centers in large cities.

Role	Importance Mean	95 % CI (Lower, Upper)	Performance Mean	95 % CI (Lower, Upper)
A	7.49	(7.48,7.51)	5.92	(5.91,5.94)
B	7.35	(7.33,7.36)	5.83	(5.82,5.85)
C	7.46	(7.44,7.47)	5.93	(5.91,5.94)
D	7.47	(7.45,7.48)	5.95	(5.94,5.96)
E	7.58	(7.57,7.60)	6.01	(6.00,6.02)
F	7.64	(7.63,7.66)	6.04	(6.03,6,06)
G	7.63	(7.62,7.64)	6.08	(6.07,6,10)
H	7.50	(7.49,7.51)	5.93	(5.92,5.94)
J	7.48	(7.47,7.49)	5.95	(5.93,5,96)

Supplementary Table S2-2. Bootstrapped mean importance and performance scores with 95% bias-corrected and accelerated (BCa) confidence intervals for nine core functions of Local Medical Centers in non-large cities.

Role	Importance Mean	95 % CI (Lower, Upper)	Performance Mean	95 % CI (Lower, Upper)
A	8.00	(7.99,8.02)	7.09	(7.08,7.10)
B	7.93	(7.92,7.94)	7.03	(7.02,7.04)
C	8.19	(8.18,8.20)	7.22	(7.22,7.23)
D	8.18	(8.18,8.20)	7.20	(7.20,7.22)
E	8.11	(8.10,8.12)	7.22	(7.21,7.23)
F	8.20	(8.19,8.21)	7.32	(7.32,7.34)
G	8.29	(8.28,8.30)	7.53	(7.52,7.54)
H	8.14	(8.12,8.15)	7.30	(7.29,7.31)
J	8.22	(8.21,8.23)	7.23	(7.22,7.24)

Supplementary Table S2-3. Bootstrapped mean importance and performance scores with 95% bias-corrected and accelerated (BCa) confidence intervals for nine core functions of Local Medical Centers in rural area.

Role	Importance Mean	95 % CI (Lower, Upper)	Performance Mean	95 % CI (Lower, Upper)
A	7.40	(7.38,7.43)	6.16	(6.14,6.18)
B	7.38	(7.35,7.40)	5.98	(5.96,6.00)
C	7.70	(7.68,7.72)	6.41	(6.39,6.43)
D	7.62	(7.59,7.64)	6.19	(6.17,6.21)
E	7.81	(7.79,7.83)	6.45	(6.43,6.47)
F	7.81	(7.79,7.84)	6.61	(6.59,6.63)
G	7.99	(7.97,8.00)	6.93	(6.91,6.94)
H	7.72	(7.70,7.74)	6.38	(6.36,6.39)
J	7.76	(7.74,7.78)	6.40	(6.38,6.42)

A=Offering professional development and training opportunities for medical staff.
B=Development of medical knowledge and innovation in disease treatment technologies
C=Provision of high-quality healthcare for local residents.
D=Delivering unmet or essential healthcare services, including advanced emergencies, psychiatric, rehabilitation, maternal, and neonatal care, which are often avoided by private healthcare providers.
E=Ensuring access to medical care for vulnerable populations—such as the economically disadvantaged, women, the elderly, persons with disabilities, and those living in remote communities.
F=Implementation of public health policies and initiatives by the national government or the Chungcheongnam-do Provincial Government.
G=Provision of a comprehensive range of services for the prevention, detection, management, and control of infectious diseases.
H=Providing coordinated care for discharged patients by fostering close collaboration and referral networks among hospitals, clinics, and community welfare services.

Supplementary Table S3-1. Differences in Role Importance across Residential Areas (Welch ANOVA, Games-Howell Test and partial η^2).

Role	Residential area	N	M (95% CI)	SD	Welch's (df)	Welch's p	Games-Howell	Patial η^2
A	Large[a]	624	7.49(7.35,7.63)	1.7	23.4(2, 992.1)	.000	$b>a^{***}, b>c^{***}$	.116
	Non-large[b]	990	8.00(7.90,8.11)	1.6				
	Rural[c]	443	7.40(7.18,7.62)	2.3				
B	Large[a]	624	7.35(7.20,7.49)	1.8	25.0(2, 988.3)	.000	$b>a^{***}, b>c^{***}$	.123
	Non-large[b]	990	7.93(7.83,8.03)	1.7				
	Rural[c]	443	7.38(7.16, 7.60)	2.3				
C	Large[a]	624	7.46(7.31, 7.60)	1.8	37.3(2, 966.3)	.000	$b>a^{***}, b>c^{***}$	.165
	Non-large[b]	990	8.19(8.09, 8.28)	1.5				
	Rural[c]	443	7.70(7.48, 7.91)	2.3				
D	Large[a]	624	7.47(7.32, 7.61)	1.9	36.1(2, 973.2)	.000	$b>a^{***}, b>c^{***}$	.169
	Non-large[b]	990	8.18(8.09, 8.28)	1.6				
	Rural[c]	443	7.62(7.40, 7.83)	2.3				
E	Large[a]	624	7.58(7.45, 7.72)	1.7	20.1(2, 1006.9)	.000	$b>a^{***}, b>c^{*}$	.079
	Non-large[b]	990	8.11(8.01, 8.21)	1.6				
	Rural[c]	443	7.81(7.61, 8.01)	2.1				
F	Large[a]	624	7.64(7.51, 7.77)	1.7	24.4(2, 1001.7)	.000	$b>a^{***}, b>c^{**}$	.095
	Non-large[b]	990	8.20(8.10, 8.30)	1.5				
	Rural[c]	443	7.81(7.62, 8.01)	2.1				
G	Large[a]	624	7.63(7.50, 7.76)	1.6	34.9(2, 1012.8)	.000	$b>a^{***}, c>a^{**}, b>c^{**}$	.126
	Non-large[b]	990	8.29(8.20, 8.38)	1.5				
	Rural[c]	443	7.99(7.81, 8.16)	1.9				
H	Large[a]	624	7.50(7.37, 7.63)	1.7	30.1(2, 1012.8)	.000	$b>a^{***}, b>c^{**}$	.122
	Non-large[b]	990	8.14(8.04, 8.23)	1.6				
	Rural[c]	443	7.72(7.53, 7.91)	2.1				
J	Large[a]	624	7.48(7.35, 7.61)	1.7	42.0(2, 993.1)	.000	$b>a^{***}, c>a^{*}, b>c^{***}$	.164
	Non-large[b]	990	8.22(8.13, 8.31)	1.5				
	Rural[c]	443	7.76(7.57, 7.95)	2.1				

M=Mean; SD=Standard Deviation; $^{*}p<.05$, $^{**}p<.01$, $^{***}p<.001$.

Supplementary Table S3-2. Differences in Role Performance across Residential Areas (Welch ANOVA, Games-Howell Test and partial η²).

Role	Residentia area	N	M(95% CI)	SD	Welch's(df)	Welch' p	Games-Howell	Patial η²
A	Large[a]	624	5.92(5.80,6.05)	1.6	113.4 (2, 1026.6)	.000	b>a***,b>c***	.457
	Non-large[b]	990	7.09(6.99,7.19)	1.6				
	Rural[c]	443	6.16(5.97,6.35)	2.0				
B	Large[a]	624	5.83(5.69,5.97)	1.8	113.6 (2, 1015.3)	.000	b>a***,b>c***	.509
	Non-large[b]	990	7.03(6.93,7.13)	1.6				
	Rural[c]	443	5.98(5.80,6.17)	2.0				
C	Large[a]	624	5.93(5.79,6.06)	1.7	127.0 (2, 1019.9)	.000	b>a***,c>a***,b>c***	.520
	Non-large[b]	990	7.22(7.13,7.32)	1.5				
	Rural[c]	443	6.41(6.24,6.58)	1.8				
D	Large[a]	624	5.95(5.82,6.08)	1.7	123.6 (2, 1010.5)	.000	b>a***,b>c***	.536
	Non-large[b]	990	7.20(7.11,7.30)	1.6				
	Rural[c]	443	6.19(5.99,6.39)	2.1				
E	Large[a]	624	6.01(5.88,6.14)	1.6	114.6 (2, 1036.0)	.000	b>a***,c>a***,b>c***	.455
	Non-large[b]	990	7.22(7.13,7.32)	1.6				
	Rural[c]	443	6.45(6.28,6.63)	1.9				
F	Large[a]	624	6.04(5.92,6.17)	1.6	131.4 (2, 1046.6)	.000	b>a***,c>a***,b>c***	.494
	Non-large[b]	990	7.32(7.23,7.42)	1.5				
	Rural[c]	443	6.61(6.44,6.78)	1.8				
G	Large[a]	624	6.08(5.97,6.20)	1.4	185.4 (2, 1085.8)	.000	b>a***,c>a***,b>c***	.616
	Non-large[b]	990	7.53(7.44,7.63)	1.5				
	Rural[c]	443	6.93(6.78,7.07)	1.6				
H	Large[a]	624	5.93(5.80,6.05)	1.6	152.4 (2, 1046.4)	.000	b>a***,c>a***,b>c***	.589
	Non-large[b]	990	7.30(7.20,7.39)	1.6				
	Rural[c]	443	6.38(6.21,6.55)	1.8				
J	Large[a]	624	5.95(5.82,6.07)	1.6	132.5 (2, 1050.2)	.000	b>a***,c>a***,b>c***	.510
	Non-large[b]	990	7.23(7.13,7.32)	1.6				
	Rural[c]	443	6.40(6.23,6.57)	1.8				

M=Mean; SD=Standard Deviation; *p<.05, **p<.01, ***p<.001

A=Offering professional development and training opportunities for medical staff.
B=Development of medical knowledge and innovation in disease treatment technologies.
C=Provision of high-quality healthcare for local residents.
D=Delivering unmet or essential healthcare services, including advanced emergencies, psychiatric, rehabilitation, maternal, and neonatal care, which are often avoided by private healthcare providers.
E=Ensuring access to medical care for vulnerable populations—such as the economically disadvantaged, women, the elderly, persons with disabilities, and those living in remote communities.
F=Implementation of public health policies and initiatives by the national government or the Chungcheongnam-do Provincial Government.
G=Provision of a comprehensive range of services for the prevention, detection, management, and control of infectious diseases.
H=Providing coordinated care for discharged patients by fostering close collaboration and referral networks among hospitals, clinics, and community welfare services.
J=Proactive measures to advance operational efficiency and institutional competence.
